# Effect of Solution Miscibility on the Morphology of Coaxial Electrospun Cellulose Acetate Nanofibers

**DOI:** 10.3390/polym13244419

**Published:** 2021-12-16

**Authors:** Ke Yan, Yao Le, Hu Mengen, Li Zhongbo, Huang Zhulin

**Affiliations:** 1 Key Laboratory of Materials Physics, CAS Center for Excellence in Nanoscience, and Anhui Key Laboratory of Nanomaterials and Nanotechnology, Institute of Solid State Physics, HIPS, Chinese Academy of Sciences, Hefei 230031, China; keyancosb@163.com (K.Y.); humengen2016@163.com (H.M.); 2 College of Light Textile Engineering and Art, Anhui Agricultural University, Hefei 230036, China; yaoleyl@126.com; 3 University of Science and Technology of China, Hefei 230026, China

**Keywords:** coaxial electrospinning, solution miscibility, core-shell structure, morphological transformation, cellulose acetate

## Abstract

Coaxial electrospinning (co-electrospinning) technique has greatly expanded the universality of fabricating core-shell polymer nanofibers. However, the effect of solution miscibility on the morphology of co-electrospun products remains unclear. Herein, different cellulose acetate (CA) solutions with high solution miscibility but distinctly different electrospinnability were used to survey the effect of solution miscibility on the co-electrospinning process. The structural characterizations show that co-electrospun products are composed of nanofibers with and without the core-shell structure. This indicates that partial solution mixing occurred during the co-electrospinning process instead of absolute no-mixing or complete mixing. Importantly, the solution miscibility also shows a significant influence on the product morphology. In particular, the transformation from nanofibers to microparticles was realized with the increase of core-to-shell flow ratio during the co-electrospinning of core electrosprayable CA/dimethylacetamide (DMAc) solution and shell electrospinnable CA/acetone-DMAc (2/1, *v*/*v*) solution. Results show that the solution miscibility exerts a significant effect on not only the formation of core-shell structure but also the product morphology. This work provides a new insight for the in-depth understanding of the co-electrospinning process.

## 1. Introduction

Electrospinning and electrospray are kindred electrohydrodynamic (EHD) techniques to produce ultrafine polymer fibers and particles [1,2], and have been extended to various fields such as nanosensors [3], drug delivery [4], tissue engineering [5], energy [6] and environment [7] applications. For the electrospinning, a continuous electrified jet is ejected from the tip of a Taylor cone, and subsequently solidified into fibers. However, if the viscoelasticity of the polymer solution can’t suppress the Rayleigh instability induced by the surface tension, the jet will break up into small droplets before its solidification, producing particles instead [8,9]. In the past two decades, newly developed coaxial electrospinning/electrospray (co-electrospinning/co-electrospray) techniques have greatly expanded the universality of fabricating polymer fibers/particles with complex structures [10,11,12]. In these processes, dissimilar solutions are usually delivered into different channels of a coaxial multichannel spinneret to achieve various structures, such as core-shell [13,14], hollow [15,16], multichannel [17], multiwall [18] or wire-in-tube [19] structures.

In a typical co-electrospinning process, the electrostatic forces focused on the shell fluid drive the core and shell fluids to form a core-shell compound Taylor cone [10,20]. Then a core-shell electrified jet is ejected from the tip of the compound Taylor cone and is subsequently solidified into core-shell fibers. For the co-electrospinning technique, there are two basic issues as discussed in detail in the review by Moghe and Gupta [21]. The first is the role of core and shell fluids in the co-electrospinning process. It has already been revealed that both the core and shell fluids play stable but different roles depending on their respective conductivity and electrospinnability [10,15,22,23,24]. The other issue is the effect of the miscibility between core and shell fluids on the formation of core-shell fibers, which has not been fully understood.

The computational [25] and experimental [26] studies both demonstrated that two miscible or partially miscible fluids usually favor a low interfacial tension, being beneficial to achieving a stable co-electrospinning process. However, there is still a divergence on whether core-shell fibers can be formed during the co-electrospinning of miscible core and shell solutions [12,21]. Some studies have shown that core-shell fibers with shape boundaries can be fabricated by co-electrospinning of miscible or even identical solutions [13,26,27]. As the electrospinning process (~1 ms) was much faster than the diffusion spreading of the boundary between two miscible solutions (0.01–1 s) no mixing took place during the co-electrospinning of two polyethylene-oxide/water-ethanol solutions with different concentrations [13]. However, it was neglected that the solution mixing might occur before the ejection of the compound jet, as the two solutions first met in the Taylor cone and kept contact for several seconds [28]. On the contrary, few researchers have also reported that the significant interdiffusion between two miscible solutions was possible during the co-electrospinning process and that it might lead to partial or even complete mixing of core and shell layers [15,28,29].

However, as far as we know, few have reported on the effect of solution miscibility and the resulting solution mixing on the morphology of co-electrospun products. It has been widely proven that a highly electrospinnable shell solution can carry out the non-electrospinnable core polymer solution or even non-polymeric liquid to form core-shell nanofibers. This means that the effect of the core fluid is modest. The morphological transformation has been typically observed in the modified co-electrospinning processes, where a solvent flow [8,30,31] or solvent saturated airflow [32] is used as the core or shell fluid instead of two viscous polymer solutions. The above limited observations indicate that a clearer understanding of the effect of solution miscibility on the co-electrospinning process and the product morphology needs to be developed through further research. It is desired that the selected core and shell solutions should have high miscibility. Meanwhile, their electrospinnability should have a difference in order to confirm the effect of solution miscibility on the product morphology.

**Figure 1:**
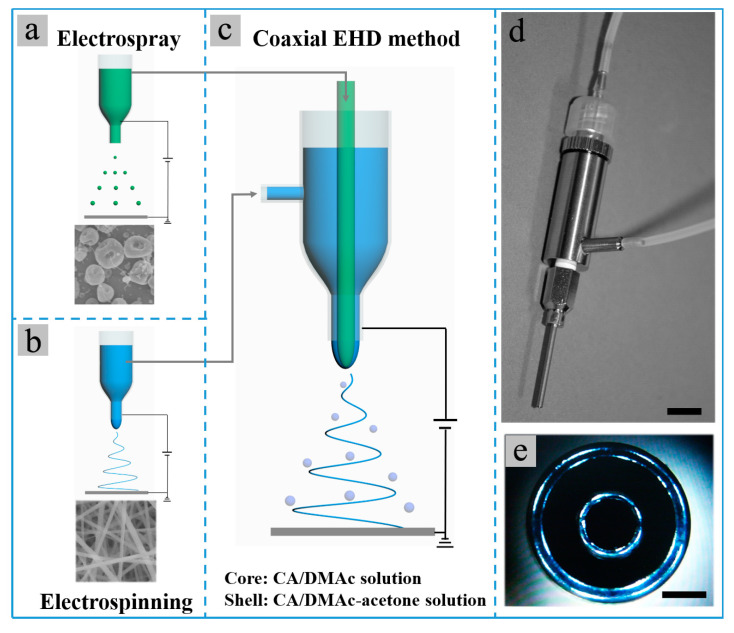
(**a**–**c**) Schematic for the (**a**) single-nozzle electrospray of CA-D solution, (**b**) single-nozzle electrospinning of CA-AD21 solution and (**c**) co-electrospinning of core CA-D solution and shell CA-AD21 solution; (**d**,**e**) photographs of the coaxial bi-channel spinneret used in the co-electrospinning experiments. The scale bars in (**d**,**e**) represent 1 cm and 0.5 mm, respectively.

Herein, we present a sample method to prepare such solution couples with high miscibility but different electrospinnability by using the same polymer but changeable solvents. Cellulose acetate (CA) is a biodegradable and eco-friendly polymer derived from natural cellulose [33]. It has been widely fabricated into nanofibers and microparticles via electrospinning and electrospraying for environmental and biological applications [33]. Furthermore, Poly (lactic-co-glycolic acid) (PLGA) is a biodegradable copolymer of poly lactic acid and poly glycolic acid, which has been widely used in drug delivery and biomaterial applications [34]. Importantly, CA dissolved in different solvents shows different electrospinnability. The CA/acetone-DMAc (2/1, *v*/*v*) solution (denoted as CA-AD21) and CA/acetone solution (denoted as CA-A) are electrospinnable, while the CA/DMAc solution (denoted as CA-D) is non-electrospinnable but shows good electrosprayability [35,36,37]. Here, two solution couples, core CA-D solution with shell CA-AD21 solution, and core CA-A solution with shell CA-D solution, were selected to survey the effect of solution miscibility, as shown in Figure 1. For comparison, core electrosprayable PLGA/DMAc (denoted as PLGA-D) solution with shell CA-AD21 solution were also selected as a solution couple with a much lower miscibility.

It has been observed that the partial mixing of core and shell solutions occurred at the tips of Taylor cones during the co-electrospinning of core electrosprayable CA-D solution and shell electrospinnable CA-AD21 solution. Furthermore, scanning electron microscope (SEM), transmission electron microscope (TEM) and fluorescence microscopy characterizations show that products with and without the core-shell structure were both produced, indicating the partial mixing rather than absolute no-mixing or complete mixing during the co-electrospinning process. In addition, the proportion of core-shell nanofibers was increased with the reduction of solution miscibility, as proved by the solution couple of core electrospinnable CA-A solution and shell electrosprayable CA-D solution. Importantly, with the increase of core-to-shell flow ratio, fiber-to-particle or particle-to-fiber transformation was achieved during the co-electrospinning of the above two highly miscible solution couples. However, only a slight morphology variation was observed during the co-electrospinning of core electrosprayable PLGA-D and shell electrospinnable CA-AD21 solutions. We concluded that the solvent diffusion from the core fluid to shell solution affected the properties of the electrified jet, such as the surface tension and solidification speed. Meanwhile, the partial solution mixing induced by high miscibility hastened this process, resulting in the significant morphological transformation. The final product morphology depended on the synergistic effect of core and shell solutions rather than the solo effect of the core or shell solution. Briefly, this work indicates that high solution miscibility and resulting partial solution mixing have a significant effect on the product morphology.

## 2. Materials and Methods

### 2.1. Materials

Cellulose acetate (Mw = 30,000) and pentanediol were purchased from Sigma-Aldrich (Shanghai, China). Poly (lactic-co-glycolic acid) (GA/LA, 50/50) was supplied by Jinan Daigang Biomaterial Co., Ltd. (Jinan, China). AgNO_3_, NaCl, Poly (vinylpyrrolidone) (PVP, M_W_ = 58,000), DMAc and acetone were obtained from Sinopharm Chemical Reagent Co., Ltd. (Shanghai, China). All chemicals were used as received without any further purification. The commercial electrospinning apparatus and coaxial bi-channel spinneret were supplied by Beijing Ucalery Technology Development Co., Ltd. (Beijing, China) and Changsha Nanoapparatus Technology Co., Ltd. (Changsha, China), respectively.

### 2.2. Preparation of Polymer Solutions

The 15% (*w*/*v*) CA-D, PLGA-D and CA-A solutions were prepared by dissolving 2.25 g polymer (CA or PLGA) into 15 mL solvent (DMAc or acetone), under magnetic stirring at 45 °C overnight. The 15% (*w*/*v*) CA-AD solutions with different solvent ratios were prepared by dissolving 2.25 g CA into 15 mL mixtures of DMAc and acetone with the acetone-to-DMAc ratio of 2/1, 1/2, 1/4 and 1/8, respectively, under magnetic stirring at 45 °C overnight. For preparing Ag nanoparticles (NPs) dispersed CA solution, Ag-NPs with the edge length ~50 nm were first prepared according to our previously reported procedures [38] and mainly involved the reduction of AgNO_3_ by pentanediol in the presence of PVP and NaCl at 155 °C. Then the as-prepared Ag-NPs were re-dispersed in the CA-D solution, followed by a vigorous ultrasonication to obtain a homogeneous solution.

### 2.3. Single-Nozzle and Coaxial Electrospinning/Electrospray Experiments

For single-nozzle electrospinning/electrospray experiments, 1 mL PTFE pipe assembled with a single-nozzle spinneret was used to load the as-prepared CA solutions. Then the electrospinning/electrospray experiments were carried out at 15 kV voltage and 12.5 cm tip-to-receiver working distance. The flow rates were all set to 0.2 mL/h. The obtained membranes were collected onto the aluminum foils and then dried in an oven overnight at 60 °C to remove the residual solvents. The environment was controlled at 20–30 °C and a humidity of ~40%.

In the co-electrospinning/co-electrospray experiments, the single-nozzle spinneret was replaced by a coaxial bi-channel spinneret (Figure 1d,e). Subsequently, the core and shell solutions were respectively fed into inner and outer channels of the bi-channel spinneret. The flow rate of the solution with higher flow rate was maintained at 0.3 mL/h in all co-electrospinning/co-electrospray experiments, while the flow rate of another solution with lower flow rate was adjusted to achieve different core-to-shell flow ratios as required. The co-electrospinning/co-electrospray experiments were carried out around 15 kV voltage and 15 cm tip-to-receiver working distance. The voltage needed to be adjusted in order to achieve stable co-electrospinning/co-electrospray processes under different core-to-shell flow ratios. The environment was controlled at 20–30 °C and humidity of ~40%.

### 2.4. Characterization

The as-prepared electrospinning/electrospray membranes were characterized by scanning electron microscope (Hitachi SU8020, Tokyo, Japan), transmission electron microscope (JEOL JEM-2010, Tokyo, Japan), inverted fluorescence microscope (Leica DMI3000 B, Wetzlar, Germany), Fourier transform infrared spectroscopy (FTIR, Nicolet Nexus, Madison, WI, USA), thermo gravimetric analysis (TGA, Mettler-Toledo TGA/DSC 3+, Greifensee, Switzerland) and X-ray diffraction (XRD, Purkinje XD6, Beijing, China). For FTIR characterization, the samples collected on Al foils were directly characterized by the FTIR spectrometer equipped with a smart diffuse reflectance accessory, a spectral resolution of 2 cm^−1^ and with 64 scans performed. For TGA characterization, samples weighing around 10 mg were measured with a heating rate of 5 ℃/min in the Ar atmosphere. Furthermore, X-ray diffraction patterns were collected over the angular range 10–80° in 20 steps of 0.03° with 4°/min scanning rate and 1 accumulation number. The XRD system was equipped with Cu Kα radiation (36kv, 20 mA, λ = 0.15406 nm) and a diffracted-beam graphite monochromator. The slit arrangement for data collection consisted of 1/6° divergence slit, 0.10 mm receiving slit and 1/2° scattering prevention slit. For the TEM characterization, a piece of membrane was immersed in ethanol and sonicated slightly to obtain the nanofiber suspension. Subsequently, the as-prepared nanofiber suspension was dropped onto a copper mesh and left to dry naturally for TEM observations. However, for directly observing the core layer, a severe sonication was applied for 2 h to break the shells of some nanofibers. And the images of Taylor cones were taken with a digital camera. In addition, the solution viscosity was tested by a rotary viscometer (Brookfield DVS+, Middleboro, MA, USA) using a S02 spindle with 20 RPM at the environment temperature of 24 °C. The measurement of the surface tension was measured with a tensiometer (Kruss DSA100, Hamburg, Germany). A needle with the diameter of 0.518 mm was used to insert a droplet of the polymer solution with the volume of 15.5 uL. Then an image was taken and subsequently analyzed by the drop shape analysis program of Pendant Drop supplied by the manufacturer to calculate the interfacial tension.

## 3. Results and Discussions

Both acetone and DMAc are good solvents for CA, meanwhile, acetone and DMAc are also highly miscible due to the adjacent values of Hildebrand solubility parameter (Table S1) [39]. Furthermore, an experiment of solvent mixing also demonstrated the high miscibility of acetone and DMAc (Figure S1). Therefore, CA, acetone and DMAc were selected to prepare highly miscible solution couples. In addition, PLGA was also selected to prepare the solution couple of CA-AD21 solution and PLGA-D solution with a much lower miscibility. Table 1 shows the viscosity and surface tension of some CA solutions and PLGA-D solution. Both the solution viscosity and surface tension increase with the rising of the proportion of DMAc in CA solutions. The surface tension of polymer solutions is close to that of the solvents used (DMAc of 32.4 and acetone of 23.7 mN/m) [35]. Furthermore, the diameter of all fibers obtained in this work was measured from 100 fibers in different regions. The corresponding diameter distribution is shown in the histograms of Figure S2, and the average fiber diameter is presented in Table 1 and Table S2.

### 3.1. Single-Nozzle Electrospray/Electrospinning of CA Solutions

**Figure 2:**
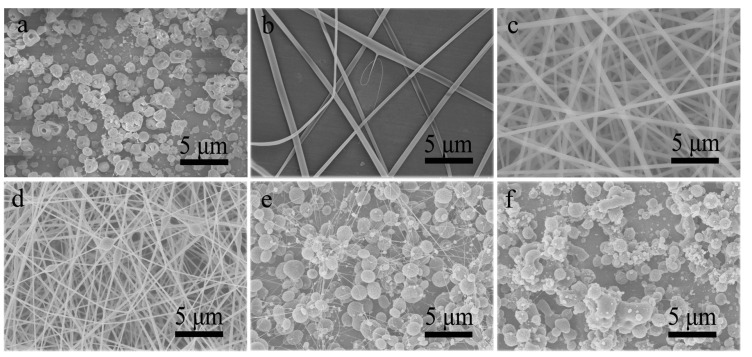
SEM images of resultant products prepared by single-nozzle electrospraying or electrospinning of (**a**) CA-D solution, (**b**) CA-A solution and (**d**–**f**) CA-AD solutions with different acetone-to-DMAc ratios of (**c**) 2/1, (**d**) 1/2, (**e**) 1/4 and (**f**) 1/8, respectively.

Frist, the single-nozzle electrospray and electrospinning of different CA solutions were performed. Although DMAc is a good solvent for CA, the CA-D solution was not electrospinnable and only irregular particles could be produced (Figure 2a), possibly owing to the high surface tension and high boiling point of DMAc [35,36]. Nevertheless, few thick fibers were fabricated by electrospinning of CA-A solution (Figure 2b), proving its electrospinnability. Unfortunately, the electrospinning process was soon interrupted by the spinneret clogging induced by the rapid evaporation of acetone. Meanwhile, the CA-AD21 solution showed good electrospinnability and could be continuously electrospun into ultrafine nanofibers (Figure 2c) as the rapid solvent evaporation was suppressed by the presence of DMAc. Furthermore, various products with different morphologies were achieved by decreasing the acetone-to-DMAc ratio in CA solutions, as shown in Figure 2d–f. Evidently, the higher the proportion of DMAc, the more likely it is to produce microparticles rather than nanofibers. Nevertheless, the fiber diameter also decreased gradually with the reduction of the acetone-to-DMAc ratio (Table 1, Figure S2a–c).

### 3.2. Co-Electrospinning of Core CA-D Solution and Shell CA-AD21 Solution

**Figure 3:**
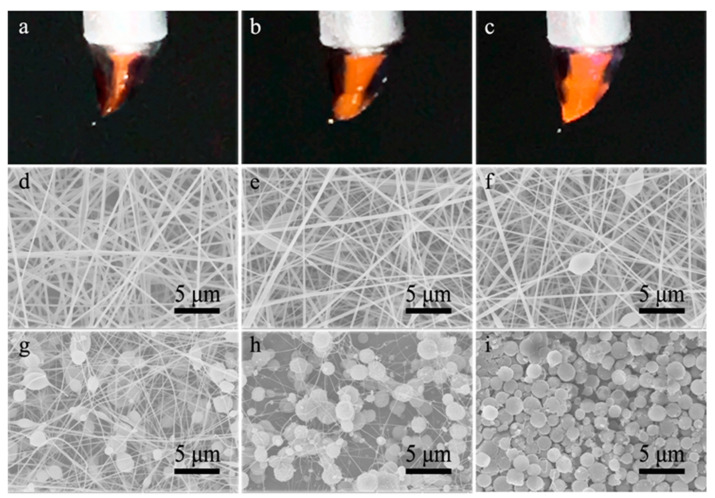
(**a**–**c**) Images of compound Taylor cones formed in the co-electrospinning of core CA-D solution and shell CA-AD21 solution under the core-to-shell flow ratio of (**a**) 1:6, (**b**) 1:1.5 and (**c**) 1:1, respectively; (**d**–**i**) SEM images of resultant nanofibers prepared under core-to-shell flow ratio of (**d**) 1:6, (**e**) 1:4.5, (**f**) 1:3, (**g**) 1:1.5, (**h**) 1:1 and (**i**) 1:0.2, respectively.

In order to observe the influence of solution miscibility on the product morphology, the electrospinnability of core and shell solutions should have a discernible difference. When the core and shell solutions are the same, the morphology of co-electrospun products should be the same or similar to that of single-nozzle electrospun products, as proved by the co-electrospinning of two well-electrospinnable CA-AD21 solutions (Figure S3). Therefore, the core solution was replaced by an electrosprayable CA-D solution, while the electrospinnable CA-AD21 solution was still selected as the shell solution. Owing to the low liquid–liquid interfacial tension [26], stable electrospinning processes proceeded successfully under a wide range of core-to-shell flow ratio from 1:6 to 1:1. Figure 3a–c show the Taylor cones formed under different core-to-shell flow ratios of 1:6, 1:1.5 and 1:1, respectively. The core-shell compound Taylor cones were formed, while partial solution mixing was observed at the tips of Taylor cones. As shown in Figure 3d–h, the increased core-to-shell flow ratio led to less and thinner nanofibers (Table S2, Figure S2d–g) accompanied with increasing and spheroidized beads. However, when the core-to-shell flow ratio exceeded 3:1, it was not easy to achieve stable co-electrospinning processes and reproducible products again. The core jet preferred to eject from the shell jet owing to its insufficient confinement, resulting in the splitting of the compound jet. Nevertheless, microparticles were occasionally produced by a careful operation (Figure 3i). As a result, the transition from bead-free nanofibers to beaded nanofibers, and further to microparticles was achieved. Evidently, the higher the flow ratio of the core solution, the more likely to produce beads and particles, which is similar to the result of previous single-nozzle electrospinning/electrospray of CA solutions.

#### 3.2.1. The Structure of Co-Electrospun CA Nanofibers

**Figure 4:**
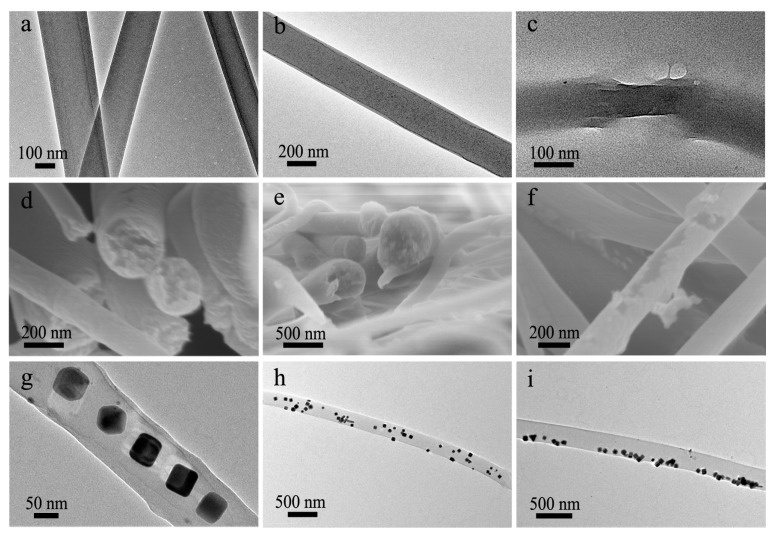
(**a**–**c**) TEM images of (**a**,**b**) intact co-electrospun nanofibers and (**c**) a co-electrospun nanofiber with broken shell; (**d**–**f**) SEM images of the (**d**,**e**) fracture surface and (**f**) surface of co-electrospun nanofibers; (**g**–**i**) TEM images of Ag-NPs loaded co-electrospun nanofibers, showing the distribution of Ag-NPs in the (**g**) central region, (**h**) whole nanofibers and (**i**) marginal area, respectively.

Owing to the formation of the same polymer of CA, FTIR, TGA, XRD and contact angle characterizations cannot distinguish the single-nozzle electrospun/electrosprayed CA fibers/particles and co-electrospun/co-electrosprayed products (Figure S4). TEM characterization is usually used to identify the core-shell structure of co-electrospun nanofibers, whereas it is also difficult to distinguish two layers in this case owing to the lack of contrast between the same material. Actually, there is no apparent contrast difference under TEM observation for many nanofibers in our case, meanwhile, slight contrast differences could also be observed for some nanofibers (Figure 4a,b). However, TEM images with such poor contrast cannot be direct evidence for the core-shell structure. To directly observe the core layer, the as-prepared nanofibers were severely sonicated to break the shell layer before TEM observation. Evidently, some nanofibers indeed have a core-shell structure (Figure 4c). Besides, the as-prepared nanofibers were immersed in liquid nitrogen and subsequently fractured for SEM observation of the fractured surface. As shown in Figure 4d, discernible core and shell layers could be observed, whereas it was difficult to clearly distinguish the core and shell layers of some nanofibers from the same sample (Figure 4e). Meanwhile, it was observed that some nanofibers were covered with an ultrathin sheath (Figure 4f), consistent with the TEM observation of Figure 4b. The shell layers were much thinner that they should be, implying the occurrence of significant solution mixing during the formation of these nanofibers. Besides, the TEM image of a bead and SEM image of particles also show their core-shell structure (Figure S5). The above characterizations show that co-electrospun products have a complicated structure that is different from conventional single-nozzle electrospinning-derived mono-phase products or co-electrospinning-derived core-shell products. The as-prepared products are composed of nanofibers both with and without the core-shell structure. The fibers without the core-shell structure could be derived from the mixing of core and shell layers. However, if the core or shell layer was interrupted in some regions, mono-layer fibers could also be obtained.

**Figure 5:**
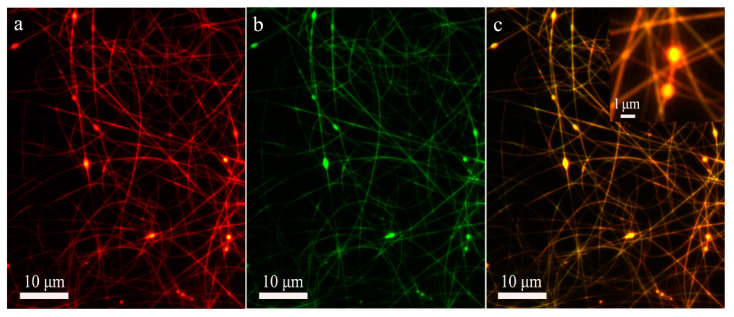
Fluorescence microscopy images of co-electrospun nanofibers produced under core-to-shell flow ratio of 1:4.5. (**a**) The shell layer; (**b**) The core layer; (**c**) The composite image of core and shell layers. The inset is an enlarged image of a selected area in (**c**), showing the core-shell structure of beads. The core and shell solutions were stained with fluorescein (green) and rhodamine (red), respectively. The yellow color is composited of green color and red color.

To confirm the formation reason of the fibers without the core-shell structure, Ag-NPs were dispersed in the core CA-D solution to show the distribution of the polymer from the core Ag/CA-D solution in the co-electrospun fibers indirectly (Figure S6). If no or slight solution mixing occurred, the Ag-NPs should be only embedded in the core region of fibers. On the contrary, if significant or complete solution mixing take place, Ag-NPs should be randomly distributed throughout the nanofibers as the diffusion of core fluid into shell solution. As shown in Figure 4g, Ag-NPs were only embedded in the central region in some cases, indicating the slight solution mixing during the formation of these fibers. Meanwhile, for some nanofibers, Ag-NPs were randomly located in the whole nanofibers (Figure 4h), implying the significant mixing of core and shell solutions. Even Ag-NPs were only occasionally embedded in the marginal area of fibers (Figure 4i), proving that the solution mixing process was relatively complex. Furthermore, the fluorescence microscopy characterization was also used to confirm whether the core or shell layers were interrupted in some regions. The corresponding images in Figure 5 show that both the shell (red color) and core (green color) polymer components are usually continuous, proving that the nanofibers without the core-shell structure were mainly derived from the mixing of core and shell layers in the local region. Above results indicate that the partial solution mixing indeed occurred during the co-electrospinning of highly miscible CA solutions, while complete mixing did not take place owing to the rapid electrified jet travel process [13].

#### 3.2.2. The Mechanism of Fiber-to-Particle Morphological Transformation

As the core-to-shell flow ratio increased, the transformation from bead-free nanofibers to beaded nanofibers, and further to microparticles was realized. Generally, the morphological transition is mainly determined by the competition of viscoelasticity against Rayleigh instability [8,21]. The former is mainly dependent on polymer chain entanglement, while the latter is mainly driven by surface tension [8,21]. For single-nozzle electrospinning, the particle-to-fiber transition can be easily achieved by increasing the polymer molecular weight [40] or concentration [9] to enhance the solution viscoelasticity, or choosing a more suitable solvent to obtained a well-electrospinnable polymer solution [35]. For co-electrospinning, Larsen et al. reported the gas jackets stabilized fiber-to-particle transition [32]. In this process, the solvent saturated jackets prevented the electrified jet from solidifying into a fiber before it destabilized naturally into droplets. Besides, Yu et al. developed a modified coaxial electrospinning method through partial replacement of the traditional shell polymer solutions by sheath solvents [30,31]. It has been found that the high boiling point solvent [30] or an excessive flow rate of the sheath solvent [31] could also induce the beads-on-a-string morphology. Nevertheless, two viscous polymer solutions were involved in our case, therefore the mechanism of fiber-to-particle transition maybe more complicated. It has been widely proven that a highly electrospinnable shell solution can serve as the “driving liquid” to carry out the non-electrospinnable core polymer solution or even non-polymeric solution to form core-shell nanofibers by viscous traction [10,15]. Therefore, as the core-to-shell flow ratio increased, such significant morphological transformation from nanofibers to microparticles is not expected in the traditional co-electrospinning process.

**Figure 6:**
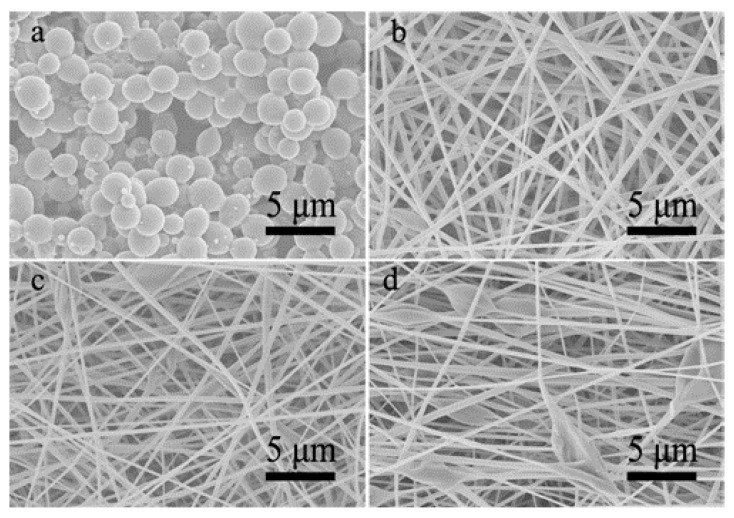
(**a**) An SEM image of single-nozzle electrospray of PLGA-D solution; (**b**–**d**) SEM images of co-electrospinning of core PLGA-D solution and shell CA-AD21 solution under core-to-shell flow ratio of (**b**) 1:3, (**c**) 1:1.5 and (**d**) 1:1, respectively.

In our case, the partial mixing of core and shell solutions occurred during the co-electrospinning process. On one hand, DMAc solvent in the core solution would mix with the shell solution to result in the property variation of the electrified jet locally [30,31]. It is assumed that DMAc solvent can clearly increase the surface tension of the shell jet due to a much higher surface tension of DMAc than acetone (32.4 vs. 23.7 mN/m) [35], which will enhance the Rayleigh instability. On the other hand, the solidification of the shell jet into a fiber should be retarded owing to the high boiling point of DMAc, which provides more time for its destabilization into beads-on-a-string shape or droplets [32]. Further, the partial solution mixing could hasten the above two effects in comparison with the solo solvent interdiffusion being beneficial to the fiber-to-particle morphological transformation. For the comparison, CA polymer in the core solution was replaced by PLGA to largely reduce the solution miscibility, while the shell CA-AD21 solution remained the same. Importantly, the PLGA-D solution is also non-electrospinnable but well electrosprayable (Figure 6a). As a result, stable co-electrospinning processes proceed successfully under the core-to-shell flow ratio from 1:3 to 1:1. Besides, the FTIR spectrum of resultant fibers shows both the characteristic peaks of PLGA and CA (Figure S7). A weak peak at 746 cm^−1^ (C-H bending), three weak peaks at 1085, 1130 and 1160 cm^−1^ (C-O stretching), and three weak peaks at 1400, 1420 and1450 cm^−1^ (CH_3_, CH_2_, and C-H deformation vibrations) were assigned to PLGA [41,42]. Meanwhile, three strong peaks at 1045, 1234, and 1365 cm^−1^ were assigned to C-O-C of the cellulose backbone, C-O stretching of the acetyl group and C-H bending vibration of CH_3_ in the acetyl group, respectively [43]. With the increase of core-to-shell flow ratio, the transformation from bead free nanofibers to fusiform-beaded nanofibers was achieved (Figure 6b–d). Obviously, the morphological variation was much smaller in comparison with the result of co-electrospinning with CA-D solution as the core solution. This result indicates that the high solution miscibility indeed facilitated the morphological transformation from fibers to particles during the co-electrospinning of CA solutions.

### 3.3. Co-Electrospinning of Core CA-D Solution and Shell CA-A Solution

**Figure 7:**
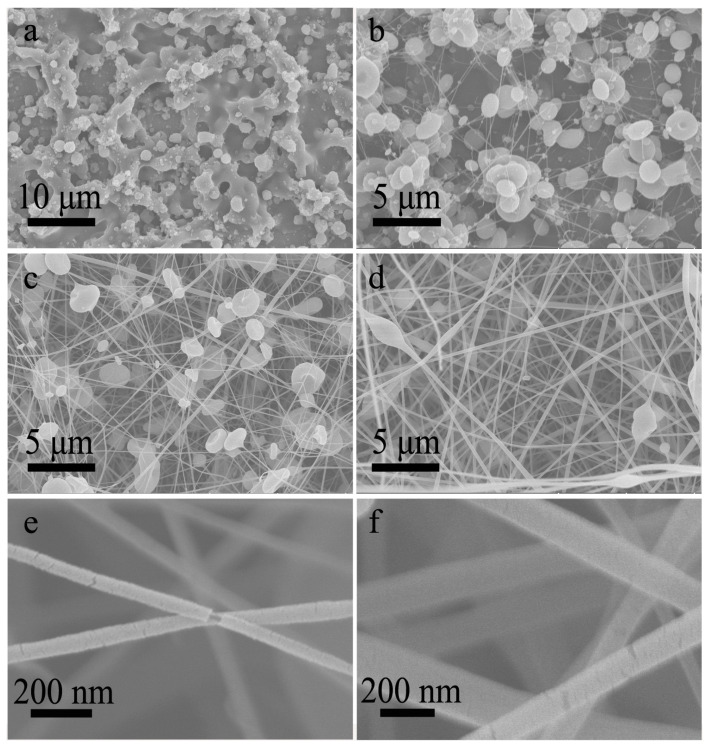
(**a**–**d**) SEM images of nanofibers prepared by co-electrospinning of core CA-A solution and shell CA-D solution under core-to-shell flow ratio of (**a**) 1:3, (**b**) 1:1.5, (**c**) 1.5:1, (**d**) 3:1, respectively. (**e**,**f**) The amplified SEM images corresponding to (**c**,**d**), showing the core-shell structure.

Further, the electrospinnable CA-A solution and electrosprayable CA-D solution were selected as the core and shell solutions, respectively. In this case, the solution miscibility was slightly reduced. A few studies have shown that an electrospinnable core solution as the spinning aid can carry out the non-electrospinnable shell solution to form core-shell nanofibers [24,25]. Nevertheless, the co-electrospinning process was not as stable as above experiments, probably owing to the insufficient electrospinnability of the shell solution. Specifically, it was not easy to obtain a stable startup process. When the high voltage was applied on the spinneret, the splitting of the compound jet occurred frequently. Through finely adjusting the working voltage, a stable co-electrospinning process could often be achieved and lasted for more than 15 min. As expected, the inversed morphological transformation from particles to fibers was also achieved. When the core-to-shell flow ratio was lower than 1:1, the resultant products were mainly composed of particles and beads (Figure 7a,b), while beaded nanofibers were fabricated when the core-to-shell flow ratio exceeded 1:1 (Figure 7c,d). In addition, the shells of resultant nanofibers were sometimes slightly broken, indicating that these nanofibers have a core-shell structure, as shown in the amplified SEM images (Figure 7e,f and Figure S8). And the proportion of core-shell nanofibers was much higher in this case, possibly owing to the reduced solution miscibility.

## 4. Conclusions

In summary, we experimentally investigated the effect of solution miscibility on the morphology as well as the structure of co-electrospun products by co-electrospinning of different CA solutions. It was found that the partial mixing of core and shell solutions occurred during the co-electrospinning of highly miscible CA-D and CA-AD21 solutions, resulting in the products composed of fibers both with and without the core-shell structure. While the complete mixing did not take place owing to the rapid electrified jet travel process. Importantly, the partial solution mixing facilitated the morphological transformation from nanofibers to microparticles with the increase of core-to-shell flow ratio. In this process, the final product morphology was dependent on the synergistic effect of core and shell solutions rather than the solo effect of the core or shell solution. In short, this work indicates that partial solution mixing occurs during the co-electrospinning of highly miscible solutions, and subsequently exerts a significant effect on not only the structure but also the morphology of co-electrospun products.

## Figures and Tables

**Table 1:** Solution properties and electrospinnability of CA and PLGA solutions.

Solution	Polymer	Solvent	Acetone-to-DMAc Ratio (*v*/*v*)	Viscosity (mPa·s)	Surface Tension (mN/m)	Product Morphology	Average Fiber Diameter (nm)
CA-D	CA	DMAc	/	1246	33.33	Particles	/
CA-A		acetone	/	370	23.62	Few fibers	574
CA-AD21		Acetone/ DMAc	2:1	544	25.52	Fibers	405
CA-AD12			1:2	/	/	Beaded fibers	218
CA-AD14			1:4	/	/	Particles and few fibers	/
CA-AD18			1:8	/	/	Particles	/
PLGA-D	PLGA	DMAc	/	825	32.82	Particles	/

## Data Availability

The data presented in this paper are available on request from the corresponding author.

## Supplementary Materials

The following are available online at https://www.mdpi.com/article/10.3390/polym13244419/s1, Table S1: Solubility parameters of CA, acetone and DMAc; Table S2: The morphologies of co-electrospun products prepared by different solution couples; Figure S1: T The photographs of mixing process between rhodamine/acetone solution and DMAc solvent, showing the high miscibility of acetone and DMAc; Figure S2: The corresponding histograms of fiber diameter. (a–c) Single-nozzle electrospinning of (a) CA-A solution, (b) CA-AD12 solution and (c) CA-AD21 solution; (d–g) Co-electrospinning of core CA-D and shell CA-AD21 solutions under the core-to-shell flow ratio of (d) 1:6, (e) 1:4.5, (f) 1:3, (g) 1:1,5; (h–i) Co-electrospinning of core CA-A and shell CA-D solutions under the core-to-shell flow ratio of (h) 1.5:1 and (i) 3:1; (j–l) Co-electrospinning of core PLGA-D and shell CA-AD21 solutions under the core-to-shell flow ratio of (j) 1:3, (k) 1:1.5 and (l) 1:1; Figure S3: (a,b) Images of compound Taylor cones formed in the co-electrospinning of two CA-AD21 solutions under the core-to-shell flow ratio of 1:3 and 1:1. (c,d) SEM images of co-electrospun CA nanofibers corresponding to (a,b). (e,f) The histograms of fiber diameter corresponding to (c,d); Figure S4: (a) FTIR characterizations of the single-nozzle electrospun CA fibers, co-electrospun CA fibers, co-electrospun beaded fibers, co-electrosprayed particles and single-nozzle electrosprayed CA particles. (b–d) TGA, XRD and contact angle of the single-nozzle electrospun CA fibers, co-electrospun CA fibers and single-nozzle electrosprayed CA particles; Figure S5: (a) A TEM image of a bead and (b) the SEM image of particles prepared by co-electrospinning of CA-D and CA-AD21 solutions; Figure S6: (a–c) SEM images of Ag-NPs loaded products prepared by co-electrospinning of core Ag-NPs-CA/DMAc solution and shell CA/acetone-DMAc (v/v, 2/1) solution under core-to-shell flow ratio of (a) 1:3, (b) 1:1.5 and (c) 1:1, respectively. (d–f) The histograms of fiber diameter corresponding to (a–c); Figure S7: (a) FTIR characterizations of the single-nozzle electrospun CA fibers, co-electrospun PLGA@CA fibers and single-nozzle electrosprayed PLGA particles; Figure S8: A SEM image of nanofibers prepared by co-electrospinning of core CA/acetone solution and shell CA/DMAc solution under core-to-shell flow ratio of 3:1, showing most nanofibers have a core-shell structure.
