# Supplementary material for: Effect of Solution Miscibility on the Morphology of Coaxial Electrospun Cellulose Acetate Nanofibers
Source: Polymers (Basel). 2021 Dec 16;13(24):4419. doi: 10.3390/polym13244419 (PMC8707229; doi:10.3390/polym13244419)
Supplement: Supplementary file 1 [file polymers-13-04419-s001.zip › polymers-1471063-supplementary.pdf]

Supporting Information

Part 1. Tables

Table S1. Solubility parameters of CA, acetone and DMAc [1]

**Solvents**	**Solubility parameter (MPa$^{1/2}$)**			
	**Hansen parameter (δt)**	**dispersion component (δd)**	**polar component (δp)**	**hydrogen bonding component (δh)**
CA	25.06	18.60	12.70	11.00
acetone	20.00	15.50	10.40	7.00
DMAc	22.7	16.80	11.50	10.20

Table S2. The morphologies of co-electrospun products prepared by different solution couples

**Core solution**	**Shell solution**	**Core-to-shell flow ratio**	**Product morphology**	**Fiber average diameter (nm)**
CA-AD21	CA-AD21	1:3	Fibers	302
		1:1	Fibers	238
CA-D	CA-AD21	1:6	Fibers	300
		1:4.5	beaded fibers	221
		1:3	beaded fibers	188
		1:1.5	beaded fibers	107
		1:1	Particles and few fibers	/
		1:0.2	Particles	/
Ag/CA-D	CA-AD21	1:3	beaded fibers	285
		1:1.5	beaded fibers	211
		1:1	beaded fibers	92
PLGA-D	CA-AD21	1:3	Fibers	326
		1:1.5	beaded fibers	238
		1:1	beaded fibers	215
CA-A	CA-D	1:3	Particles	/
		1:1.5	Particles and few fibers	/
		1.5:1	beaded fibers	91
		3:1	beaded fibers	205

Part 2. Figures

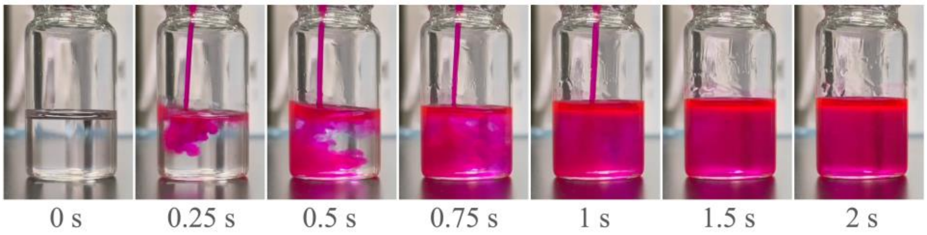

Figure S1. The photographs of mixing process between rhodamine/acetone solution and DMAc solvent, showing the high miscibility of acetone and DMAc.

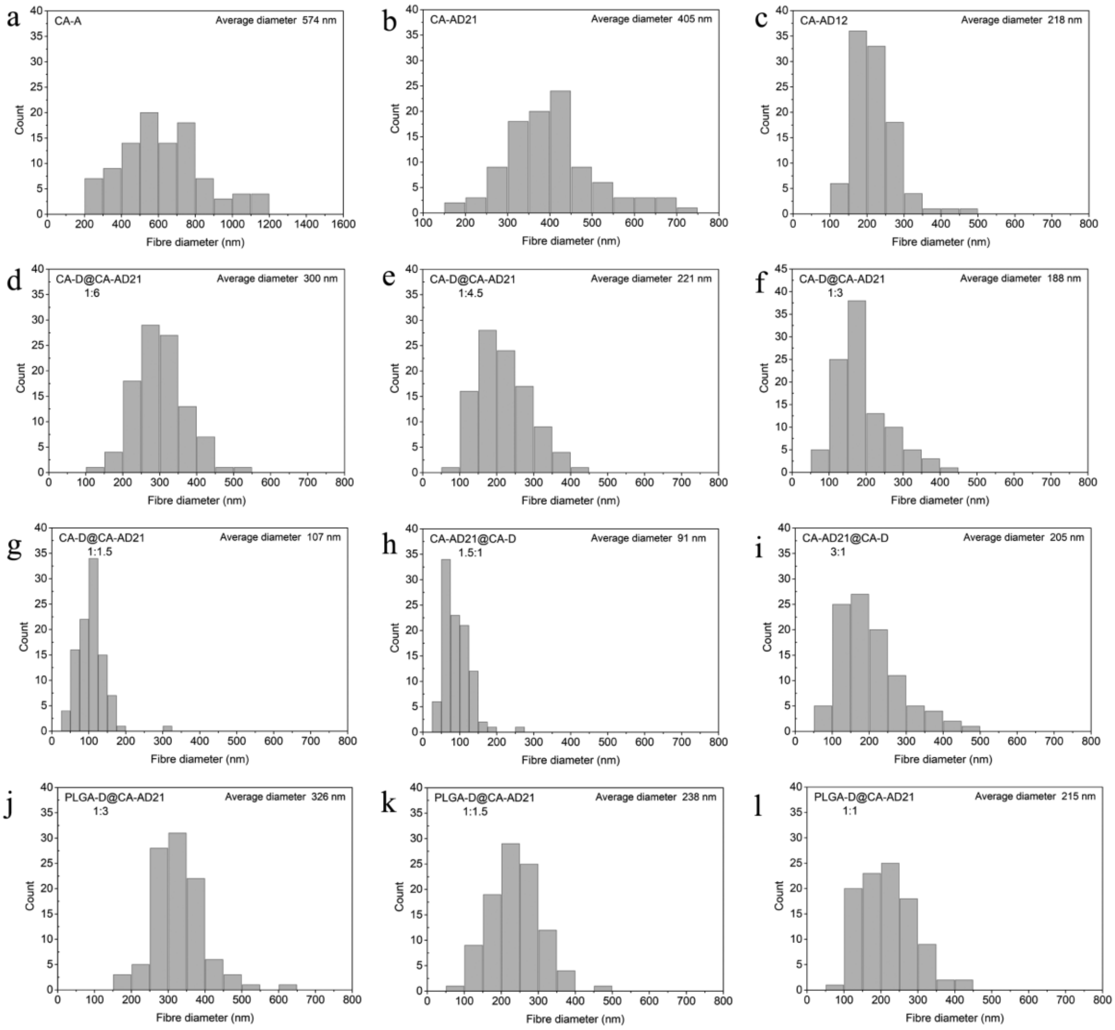

Figure S2. The corresponding histograms of fiber diameter. **(a)-(c)** Single-nozzle electrospinning of **(a)** CA-A solution, **(b)** CA-AD12 solution and **(c)** CA-AD21 solution; **(d)-(g)** Co-electrospinning of core CA-D and shell CA-AD21 solutions under the core-to-shell flow ratio of **(d)** 1:6, **(e)** 1:4.5, **(f)** 1:3, **(g)** 1:1,5; **(h-i)** Co-electrospinning of core CA-A and shell CA-D solutions under the core-to-shell flow ratio of **(h)** 1.5:1 and **(i)** 3:1; **(j)-(l)** Co-electrospinning of core PLGA-D and shell CA-AD21 solutions under the core-to-shell flow ratio of **(j)** 1:3, **(k)** 1:1.5 and **(l)** 1:1.

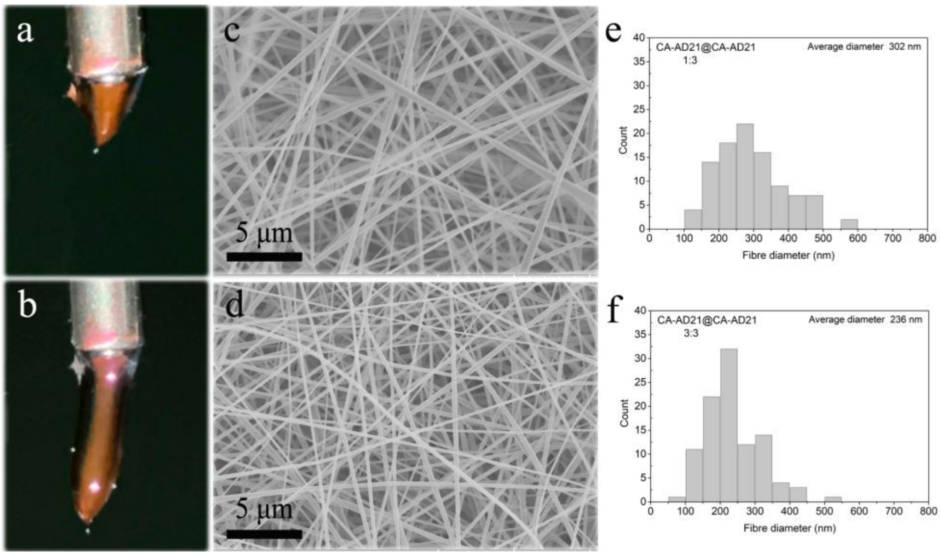

Figure S3. (a, b) Images of compound Taylor cones formed in the co-electrospinning of two CA-AD21 solutions under the core-to-shell flow ratio of 1:3 and 1:1. **(c, d)** SEM images of co-electrospun CA nanofibers corresponding to **(a, b)**. **(e, f)** The histograms of fiber diameter corresponding to **(c, d).**

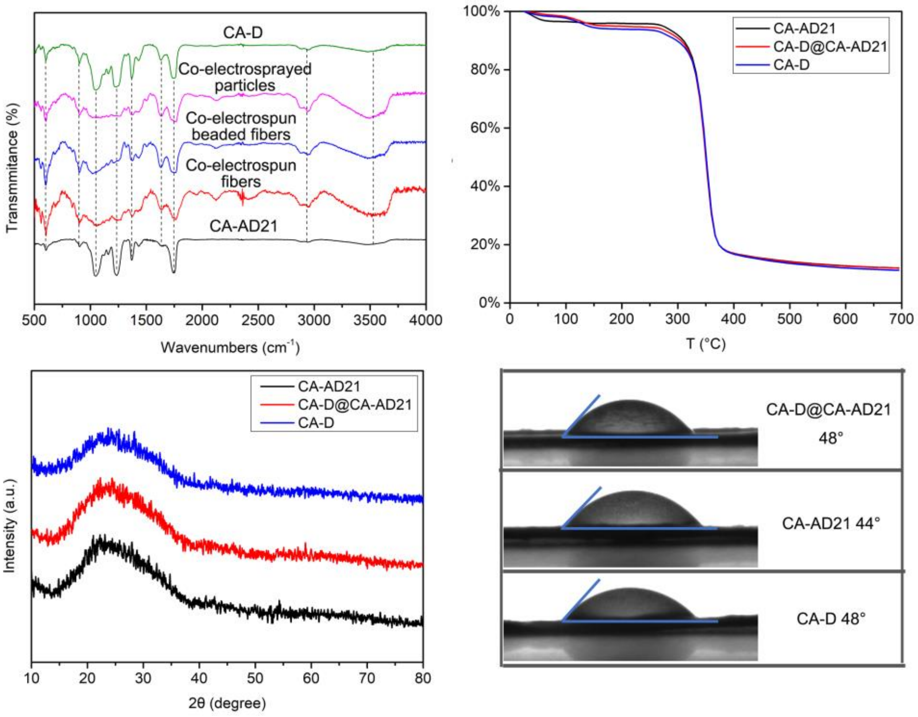

Figure S4. (a) FTIR characterizations of the single-nozzle electrospun CA fibers, co-electrospun CA fibers, co-electrospun beaded fibers, co-electrosprayed particles and single-nozzle electrosprayed CA particles. **(b)-(d)** TGA, XRD and contact angle of the single-nozzle electrospun CA fibers, co-electrospun CA fibers and single-nozzle electrosprayed CA particles.

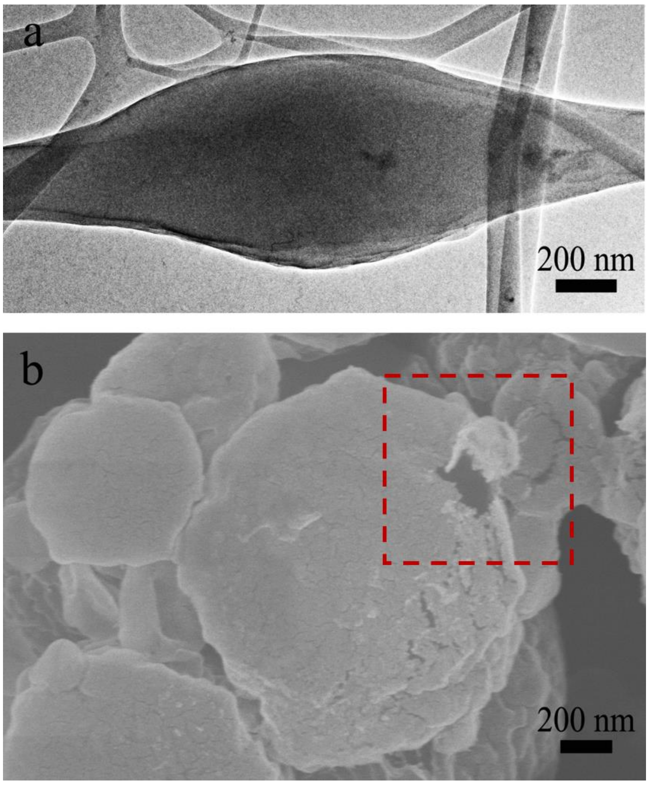

Figure S5. (a) A TEM image of a bead and **(b)** a SEM image of particles prepared by co-electrospinning of CA-D and CA-AD21 solutions.

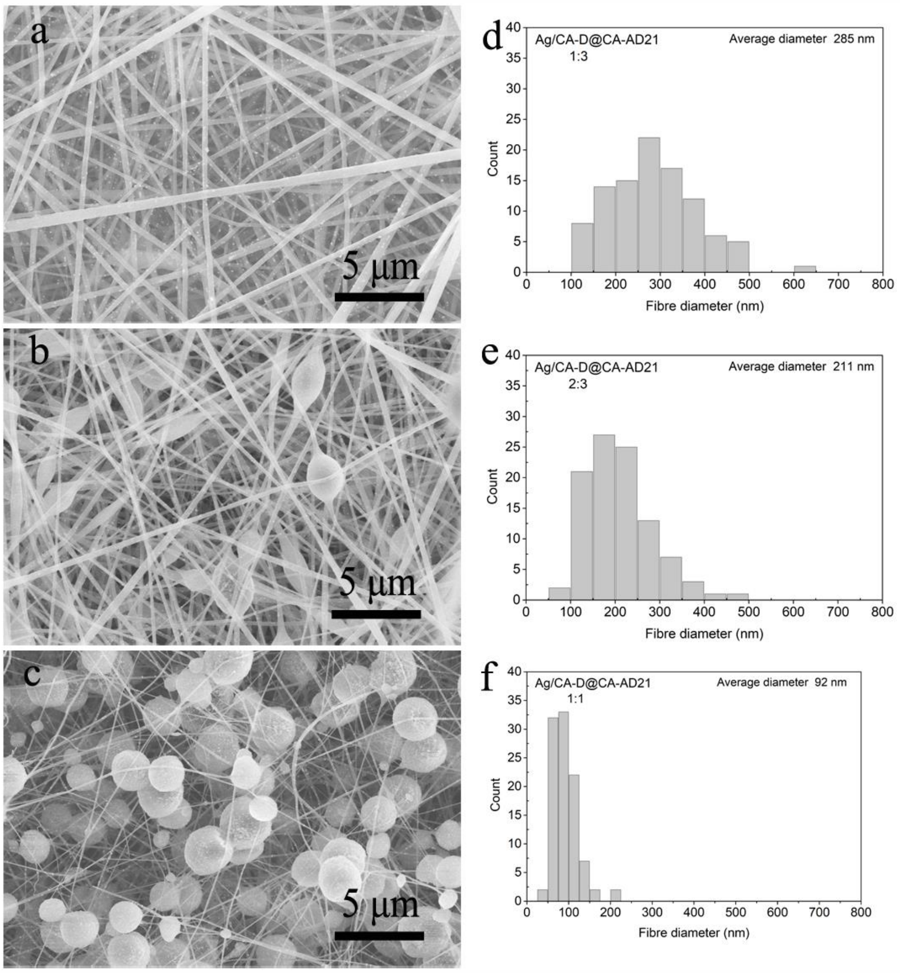

Figure S6. (a)-(c) SEM images of Ag-NPs loaded products prepared by co-electrospinning of core Ag-NPs-CA/DMAc solution and shell CA/acetone-DMAc (v/v, 2/1) solution under core-to-shell flow ratio of **(a)** 1:3, **(b)** 1:1.5 and **(c)** 1:1, respectively. **(d)-(f)** The histograms of fiber diameter corresponding to **(a)-(c)**.

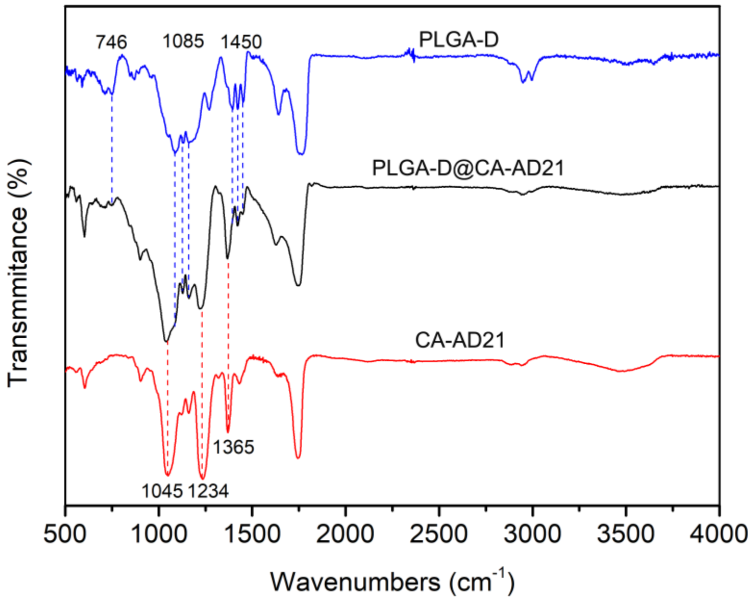

Figure S7. (a) FTIR characterizations of the single-nozzle electrospun CA fibers, co-electrospun PLGA@CA fibers and single-nozzle electrosprayed PLGA particles.

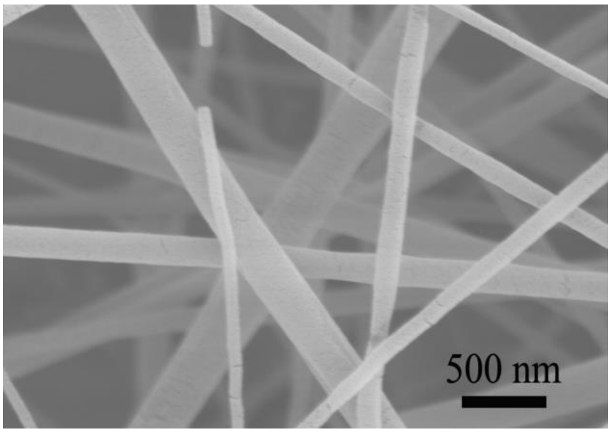

Figure S8. A SEM image of nanofibers prepared by co-electrospinning of core CA/acetone solution and shell CA/ DMAc solution under core-to-shell flow ratio of 3:1, showing most nanofibers have a core-shell structure.

Reference

[1] Haas D.; Heinrich S.; Greil P. Solvent control of cellulose acetate nanofibre felt structure produced by electrospinning. J. Mater. Sci. **2010**, 45, 1299-1306.
